# Variations in Payment Allocation to Persons Living With Cognitive Impairment and Study Partners

**DOI:** 10.1093/geroni/igab046.808

**Published:** 2021-12-17

**Authors:** Miranda McPhillips, Junxin Li, Justine Sefcik, Darina Petrovsky, Glenna Brewster, Nancy Hodgson, Nalaka Gooneratne

**Affiliations:** 1 University of Pennsylvania, University of Pennsylvania, Pennsylvania, United States; 2 Johns Hopkins University, Baltimore, Maryland, United States; 3 Drexel University, College of Nursing and Health Professions, Philadelphia, Pennsylvania, United States; 4 Rutgers University, Philadelphia, Pennsylvania, United States; 5 Nell Hodgson Woodruff School of Nursing Emory University, Atlanta, Georgia, United States; 6 University of Pennsylvania, School of Nursing, Philadelphia, Pennsylvania, United States; 7 University of Pennsylvania School of Medicine, Philadelphia, Pennsylvania, United States

## Abstract

There is a paucity of research focused on monetary incentives for recruiting dyads (participants with cognitive impairment and study partners) into research. Our objective was to evaluate if two different variations in allocating compensation among dyads changed consent rates in one clinical trial, Memories2. This trial is evaluating cognitive and functional outcomes of obstructive sleep apnea treatment in patients with amnestic mild cognitive impairment (aMCI). Prior to phone screening, participants were randomly assigned to one of two groups (1) $200 to participant with aMCI or (2) $100 to participant with aMCI and $100 to study partner at consent visit. Allocating all the payment to the participant with aMCI yielded a 2.6% consent rate, while splitting the payment yielded at 1.7% consent rate. We will also discuss how demographic factors affected consent decision by group. This study provides insight into novel strategies that may enhance enrollment of dyads into clinical trials.

